# Oleuropein-Laded Ufasomes Improve the Nutraceutical Efficacy

**DOI:** 10.3390/nano11010105

**Published:** 2021-01-04

**Authors:** Maria Chiara Cristiano, Francesca Froiio, Antonia Mancuso, Donato Cosco, Luciana Dini, Luisa Di Marzio, Massimo Fresta, Donatella Paolino

**Affiliations:** 1 Department of Experimental and Clinical Medicine, University “Magna Græcia” of Catanzaro, 88100 Catanzaro, Italy; mchiara.cristiano@unicz.it (M.C.C.); f.froiio@unicz.it (F.F.); 2 Department of Health Sciences, University of Catanzaro “Magna Graecia”, 88100 Catanzaro, Italy; antonia.mancuso@unicz.it (A.M.); donatocosco@unicz.it (D.C.); fresta@unicz.it (M.F.); 3 Department of Biology and Biotechnology “Charles Darwin”, Sapienza University of Rome, 00185 Rome, Italy; luciana.dini@uniroma1.it; 4 Department of Pharmacy, University of Chieti-Pescara “G. d’Annunzio”, 66100 Chieti, Italy; luisa.dimarzio@unich.it

**Keywords:** ufasomes, linoleic acid, oleic acid, oleuropein, antioxidant activity, unsaturated fatty acid liposomes

## Abstract

Ufasomes are unsaturated fatty acid liposomes made up of oleic and linoleic acids, natural components required in various biological processes. This kind of nanocarrier is characterized by a simple and dynamic structure and is able to improve the bioavailability of unsaturated fatty acids. The aim of this investigation was to evaluate ufasomes as natural compound delivery systems to deliver oleuropein and improve its antioxidant activity. Oleuropein is a phenolic compound mainly present in olives and olive oil, with several biological properties, such as the antioxidant activity. However, to improve their biological activity, antioxidant compounds should be able to cross cell membranes and uniformly incorporate in cells. Because of the great similarity between their constituents and cell membranes, ufasomes could be advantageous carriers for oleuropein delivery. The physico-chemical characteristics of ufasomes were investigated. A regular shape was shown by transmission electron microscopy studies, while the mean sizes were dependent on the ufasomes composition. In vitro studies highlighted that empty ufasomes did not lead to cell mortality at the tested concentrations and a good carrier internalization in CaCo-2 cells, further studies in vitro studies demonstrated that oleuropein-loaded ufasomes were able to enhance the antioxidant activity of the free active substance making this carrier a suitable one for nutraceutical application.

## 1. Introduction

Today, “nutraceutical” is a fashionable term that attracts a lot the consumer and patient. In reality, it is not a recently coined term, but it was introduced for the first time in 1986 by Stephen De Felice to indicate a natural product, often as food, that elicit a medical or health benefit, such as the prevention and/or the treatment of a diseases [1]. The term nutraceuticals was born from the combination of the words “nutrition” and “pharmaceutical” [2], highlighting the coexistent of two different approaches.

Oleuropein is an example of natural product characterized by nutraceutical properties and considered as “super functional food”. It is a secoiridoid precursors of HT (3,4-dihydroxyphenylethanol (3,4-DHPEA)) and it is mainly responsible for the bitter taste of olive leaves [3]. The leaves of olive tree are waste material, and their use for the extraction of oleuropein can also be important in terms of environmental impact. 

**Figure 1:**
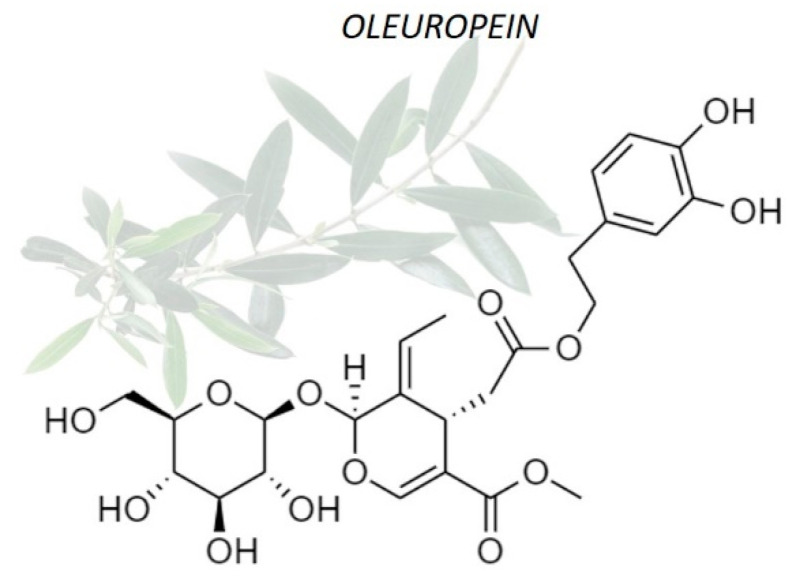
Chemical structure of oleuropein. The *o*-dihydroxy (catechol) groups present in its chemical structure confer antioxidant properties of oleuropein.

The current study of oleuropein benefits is in continuous deepening, thanks to the multiple applications of this healthy product of natural origin. Several research groups evidenced that oleuropein plays a key role in prevention and for treating of a large number of diseases and pathological conditions [4]. For example, it was found that oleuropein is characterized by antiviral power against some virus [5]; hypoglycemic effects useful in the diabetes treatment [6]; anti-aging effects with a mechanism similar to that of vitamin E [7]; anti-inflammatory effects by inhibiting lipoxygenase activity [8] for example on colon tissue [9]; neuroprotector effect preventing hypoxia and ischemia [4]; and, no less important, it seems to be characterized by a certain antitumoral activity due to its ability to inhibit the cell proliferation [10]. Many of the biological properties of oleuropein may be related to their antioxidant and free-scavenger ability [11,12]. In particular, oleuropein has been demonstrated to ameliorate oxidative damage by scavenging free radicals in vivo [13]. The potent antioxidant activity of oleuropein seems to be correlated with its hydroxyl groups, which avoids oxidation by hydrogen donation [14], carrying out a cytoprotective effect against oxidative stress such as H_2_O_2_, as already demonstrated [15] (Figure 1).

Unfortunately, as many “super functional foods” and nutraceuticals, oleuropein is characterized by a low solubility, high sensitivity to environmental conditions, and bad sensorial features [16], in fact as already mentioned, oleuropein is responsible for the bad taste of olive leaves [17]. These problems reduce the possibility of exploiting the several nutraceutical properties of oleuropein. In this scenario, nanosystems delivering active compounds are considered a new strategy to allow a more advantageous and suitable administration of oleuropein and some research groups have already been proposed the use of liposomes [16,18] and nanostructured lipid carriers (NLC) [19,20] to deliver and to protect the oleuropein, but to the best of our knowledge no one has investigated the potential use of ufasomes for oleuropein delivery. 

Ufasomes was proposed for the first time in 1973 by Gebicki and Hicks [21] as “*unsaturated fatty acid vesicles*” characterized by a closed lipid bilayer membrane, and they are included in the category of fatty acid vesicles, made up of fatty acid and their ionized species [22,23]. Although to date the literature concerning ufasomes is scarce, some research groups have prepared fatty acid vesicles using different saturated, unsaturated and highly unsaturated fatty acid, such as octanoic acid [24] oleic acid, linoleic acid, and docosahexaenoic acid [25]. The main components of ufasomes are normally oleic acid (*cis*-9-octadecenoic acid) and linoleic acid (*cis*, *is*-9,12-octadecadienoic acid), that are unsaturated fatty acids and their use leads to many advantages. In particular, compared with their better-known precursor liposomes, the ufasomes present a dynamic nature due to the presence of single-chain amphiphiles in their composition, which makes them more versatile by placing them between conventional nanosystems formed from double-chain amphiphiles and micelles formed from single-chain surfactants [22]. Moreover, ufasomes are characterized by good biocompatibility, easy bioavailability of constituents, abundant for example in olive oil [26], and generally by a simple assembly strategy [23]. 

The ufasomes, due to their natural composition, can be considered nutraceuticals themselves. The oleic and linoleic acid contained in their composition can immediately be available to target cells, showing their pharmacological activity. It is in fact known that oleic acid, at particular doses, is a strategic component in cancer prevention [27], as well as being able together with linoleic acid to inhibit cell proliferation in several cancer, such as prostate carcinoma [28] and colon cancer [29]. 

Basing on this evidence, the aim of this research work was to investigate ufasomes as potential nanosystems for oleuropein delivery, in order to combine the benefits of unsaturated fatty acids and entrapped nutraceutic, and to improve cell-nanosystems interaction and antioxidant activity of oleuropein on CaCo-2 cell model. The ufasomes were characterized from a physico-chemical and technological points of view, and in vitro studies were performed to evaluate the protective effects of oleuropein-loaded ufasomes when cell model was subjected to oxidative stress.

## 2. Materials and Methods

### 2.1. Materials

Phospholipon^®^ 90G (PL90G), containing 93.63% of phosphatidylcholine, was provided by Lucas Meyer C., Hamburg, Germany. Oleic acid, linoleic acid, oleuropein, 3-[4,5-dimethylthiazol-2-yl]-3,5-diphenyltetrazolium bromide salt (MTT), and dimyristoyl- phosphoethanolamine sulforhodamine B (DMPE-rhodamine) were purchased from Sigma-Aldrich (Darmstadt, Germany). Double distilled water was used to prepare all samples. Oleuropein release tests were performed by Spectra/Por cellulose membranes, with a cut off of 10,000 Da, which were provided by Spectrum Laboratories Inc (Rancho Dominguez, CA, USA). The human colorectal adenocarcinoma cell line, CaCo-2, were provided by the Istituto Zooprofilattico of Modena and Reggio Emilia (Reggio Emilia,) and used for the in vitro evaluation of the system toxicity. Trypsine/EDTA, culture medium (DMEM) and fetal bovine serum (FBS) were obtained from GIBCO (Invitrogen Corporation, Paisley, UK). All substances and solvents used in this experimental investigation were highly pure and require no further purification procedures.

### 2.2. Preparation of Samples

Three batches of two different formulations (A and B) of ufasomes were prepared by using a mixture of oleic acid, linoleic acid and Phospholipon^®^ 90G (molar ratio 1:1:0.5) or a mixture of oleic and linoleic acid (molar ratio 1:1). A new preparation method was developed in Advanced Drug Delivery Lab of University “Magna Graecia” of Catanzaro. In detail, the lipid mixture was suspended in distilled water and was then homogenized at 15,000 rpm for 20 min using an Ultra-Turrax T25 equipped with an S25 N-8G homogenizing probe (IKA-WERKE). The homogenizing cycle was repeated 3 times with 5 min at rest, and the preparation phase was carried out maintaining the sample in an ice bath. To achieve oleuropein-loaded ufasomes and rhodamine-labeled ufasomes, oleuropein (10 µM and 100 µM) or DHPE-rhodamine (0.1% molar) were added in the lipid mixture. Finally, the formulations were kept under continuous stirring for 20 min to stabilize. In Table 1 is presented the specific qualitative-quantitative composition of samples.

### 2.3. Physico-Chemical and Technological Characterization of Carriers

The mean sizes, polydispersity index and Z-potential of samples were evaluated by the dynamic light scattering technique using the Zetasizer Nano ZS (Malvern Instruments Ltd., Worchestershire, UK). This instrument is a spectrophotometer equipped with a rated output of 4.5 mW laser diode, operating at 670 nm with a backscattering angle of 173°. The dielectric constant (80.4), the medium refractive index (1.330) and the medium viscosity of 1.0 mPa × s was set before the analysis. The different samples were put in quartz cuvettes and submitted for analysis at room temperature. Ten different measurement were performed for each sample and results represent their average value ± standard deviation. The Smoluchowsky constant F (Ka) of 1.5 was used to obtain Z-potential values as a function of the electrophoretic mobility of the nanoparticles. 

The stability of the vesicular systems was evaluated by Turbiscan Lab^®^ Expert (Alfatest S.r.l., Rome, Italy) as a function of back scattering (∆bs) and transmission (∆t) profiles of samples. Cylindrical glass tubes were used for analysis and a Turby Soft 2.0 software (Alfatest S.r.l., Rome, Italy) were used to process the results. Moreover, Turbiscan Stability Index (TSI) profiles versus time were used to compare the different formulation in terms of stability. The measurements were performed at 24 ± 1 °C for 1 h [30]. *TSI* is a statistical parameter obtained using the following equation:

(1) $$TSI=\sqrt{\frac{\sum_{i=1}^{n}{\left(x_{i}-x_{bs}\right)}^{2}}{n-1}}$$

where *x_i_* is the mean backscattering for each minute of measurements; *x_bs_* is mean of *x_i_*; and *n* is the number of scans [31].

### 2.4. Transmission Electron Microscopy (TEM)

The transmission electron microscope (Philips, Eindhoven, The Netherlands) was used to evaluate the morphology of the vesicular carriers. Samples were analyzed at 500 nm. A drop of sample was placed onto standard TEM carbon-coated Cu-grids. The sample was dried and treated with a suitable contrast medium, uranyl acetate, for 2 min and washed with distilled water [32].

### 2.5. Entrapment Efficacy of Samples

The amount of entrapped oleuropein was evaluated by centrifugation using Amicon Ultra 3000 Da MWCO-Merck Millipore (Molsheim, France). The samples were centrifugated at 4000 rpm at room temperature for 20 min by a centrifuge Eppendorf 5810. The pellet and the supernatant were divided and the amount of non-untrapped compound was evaluated by HPLC. Due to the amphiphilic characteristic of oleuropein, the encapsulation efficiency (EE%), which represents the amount of encapsulated compound in the vesicular systems, was calculated in duplicate, both on the supernatant and on the pellet. In detail, the encapsulation efficiency, evaluated on supernatant, was indicated as the difference between the amount of oleuropein used for the colloidal systems preparation and the non-encapsulated amount, present in the supernatant (Equation (2)). While, the EE, evaluated on pellet, was indicated as the ratio between the amount of active substance present in the pellet and the total amount of oleuropein used for the preparation (Equation (3)).

The EE% was evaluated according to the following equations:

(2) $$\mathrm{EE}\%=\;\frac{\mathrm{PA}-\mathrm{PN}}{\mathrm{PA}}\times 100$$

(3) $$\mathrm{EE}\%=\;\frac{\mathrm{PN}}{\mathrm{PA}}\times 100$$

where PA is the amount (mg) of the active substance added during the preparation of samples and PN was the amount (mg) of the non-entrapped active substance [33].

### 2.6. Oleuropein Release Profiles

Dynamic Franz diffusion cells (LGA, Berkley, CA, USA) were used to evaluate the release profiles of oleuropein from ufasomes. A synthetic cellulose membrane, previously hydrated in bidistilled water for 1 h, was interposed between the donor and the receptor compartment of this system. The receptor (lower part) was filled with an ethanol/water mixture (20:80 *v*/*v*) and maintained under constant stirring at 360 rpm, with a small magnetic stirring bar, to guarantee the system homogeneity. Moreover, 200 µL of each formulation were put in the donor (upper part) to evaluate its permeation profile through the synthetic membrane. The diffusion area of these Franz cell was of 0.75 cm^2^ and the nominal receiving volume of 4.75 mL. The duration of experiment was 24 h and at predetermined time intervals, an aliquot of receptor phase was automatically withdrawn and replaced with fresh solution. During the entire experiment, the temperature was maintained at 37 ± 0.1 °C (GR 150 thermostat, Grant Instruments Ltd., Cambridge, UK) [34]. The withdrawn samples were collected and analyzed by HPLC to evaluate the release profiles of active substances.

### 2.7. HPLC Analysis

Samples of oleuropein derived from entrapment efficacy and release profile were analyzed using HPLC (A Jasco PU-1580 intelligent HPLC pump, Tokyo, Japan). The chromatographic system was equipped with a UV photodiode detector (multiwavelength) (Jasco MD 1510 diode, Tokyo, Japan). The column C18 in reversed-phase (250 by 4.60 mm–5.0 µm), was maintained at room temperature. Other chromatographic conditions were as follows: Mobile phase was a 25:75 (*v*/*v*) mixture of acetonitrile:water and flow rate was 1 mL/min [20]. The UV detection wavelength was 230 nm. 

### 2.8. Cell Cultures and In Vitro Cytotoxic and Antioxidant Evaluation 

The human colorectal adenocarcinoma cell line, CaCo-2, were incubated in plastic culture dishes (100 by 20 mm) using DMEM medium, containing penicillin (100 UI/mL), streptomycin (100 µg/mL), amphotericin B (250 µg/mL), and FBS (10% *v*/*v*), until reaching 80% confluence [35]. After washing and eliminating all waste substances, the trypsin was used to detached cells and, following centrifugation and resuspension, they were placed in 96-well culture dishes at a density of 10,000 cells/0.2 mL, for all in vitro experiments. 

The in vitro cytotoxic evaluation of formulations was performed on CaCo-2 in as a function of lipid concentration of ufasomes and the results were expressed in terms of cell viability (%). The study was carried out by using MTT test and cell viability was calculated (Equation (4)), as previously reported [36].

Cell viability (%) = AbsT/AbsC × 100(4)

where AbsT is the absorbance of treated cells and AbsC the absorbance of untreated cells (control). The ELISA microplate reader (BIO RAD, xMark™ Microplate Absorbance Spectrophotometer, Hercules, CA, USA) at λabs 570 nm and λem 670 nm was used to study the absorbance values of all the analyzed samples.

The antioxidant activity of free oleuropein and oleuropein-loaded ufasomes was evaluated by means lactic hydrogenase (LDH) assay and MTT test; the results were expressed in terms of LDH release (%) and cell viability (%), respectively. The LDH assay is a method that correlates the LDH release with the cellular membrane alteration and cell disruption [37]. In detail, CaCo-2 cells placed in 96-well culture dishes where before treated with increased concentrations of oleuropein, both in free form and as loaded ufasomes for 24 h, and then they were incubated with hydrogen peroxide (700 µM) for 1 h to induce an oxidative stress. The antioxidant protective effect of compounds was evaluated using a Pierce LDH cytotoxicity assay kit and following its specific protocol. LDH release was analyzed by spectrophotometer in the cultured medium at λ = 680 nm and λ = 490 nm, and calculated as reported in Equation (5).

(5) $$LDH\;released\left(\%\right)\;=\;\;\frac{LDHcompounds-LDHspontaneous}{LDHmaximum-LDHspontaneous}\times 100$$

where *LDHcompounds* represents the absorbance of LDH released from treated cells; *LDHspontaneous* represents activity the LDH released from CaCo-2 without any stimulus; and *LDHmaximum* represents LDH activity released form cells after lysis. The results were reported as the average of three different experiments ± standard deviation.

### 2.9. Confocal Laser Scanning Microscopy (CLSM)

The interaction between the CaCo-2 cell line and the vesicular carrier was evaluated by the Confocal laser scanning microscopy (CLSM, Leica TCS SP2 MP, Buccinasco, Milan, Italy). The cells were plated in 6-well culture dishes (4 × 10^4^ cells/mL) containing a sterile slide. The plates were incubated for 24 h and then, they were treated with the rhodaminated carrier formulations (rhodamine-DMPE) for different incubation times. After this incubation time, PBS was used to wash each well (three times) in order to remove excess colloidal suspension. An ethanolic solution (70% *v*/*v*) was used to fix the cells on the sterile slide. Each well was treated with 1 mL of Hoechst solution (0.01 µg/mL) for 30 min to highlight the nuclei, so they were washed with PBS for three times. The plates were stored at 4 °C until the analysis. Just before the analysis, the slides were placed on coverslips, using a glycerol solution (70% *v*/*v*) to remove the air inside and they were fixed using transparent glue [38]. 

A scanning laser microscope (Leika TCS SP2 MP, Buccinasco, Milan, Italy) was used for the analysis operating at a λ_exc_ = 560 nm and a λ_em_ = 580 nm for the rhodamine probe and at a λ_exc_ = 405 nm and a λ_em_ = 460 nm for the Hoechst probe. A scan resolution up to 1024 × 1024 pixels with laser beam Ar/Kr of 75 mW, equipped with a TRITC analyzed filter, was used for experiments. For viewing the samples, an oil immersion objective 63× was used.

### 2.10. Statistical Analysis

Statistical data analysis was performed by one-way ANOVA. Bonferroni *t*-test was used to confirm the results. A *p* value ≤ 0.005 was considered statistically significant. All values are reported as the average ± standard deviation. 

## 3. Results

### 3.1. Physico-Chemical and Technological Characterization of Vesicular Carriers

**Figure 2:**
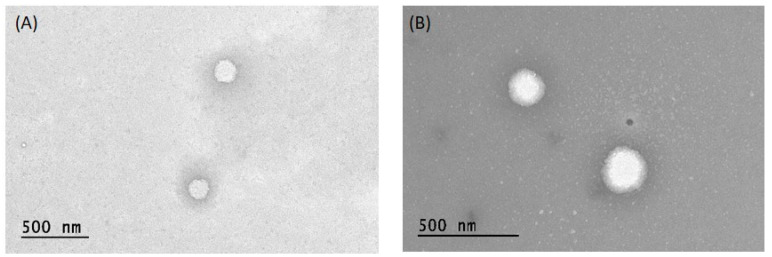
Transmission electron microscopy (TEM) of (**A**) and (**B**) samples.

During the design and planning of a new pharmaceutical system, or during the refinement and investigation of an already existing system, it is important to evaluate the physico-chemical characteristics that may arise from any changes made. For this reason, an in-depth chemical-physical and technological-formulation characterization was required. Two empty ufasome formulations, characterized by different molar ratio of lipid components, were prepared and analyzed in this study. The idea to consider the unsaturated fatty acids for this study is certainly due to their usefulness in various biological process and in the prevention of certain pathological events such as oxidative stress and inflammation [21]. In detail, a morphological characterization of samples was achieved by transmission electron microscopy (TEM) analysis, to understand the shape assumed by the carrier following the arrangement of the used components. For our unsaturated fatty acid-nanosystems the lipophilic components self-arrange by giving a spherical and regular shape to the ufasomes as demonstrated in Figure 2. The obtained shape was very similar to that of the most common vesicular carriers, such as liposomes and ethosomes [39,40], and it is consistent with the closed lipid bilayer structure previously proposed and showed by Gebicki et al. [21].

**Figure 3:**
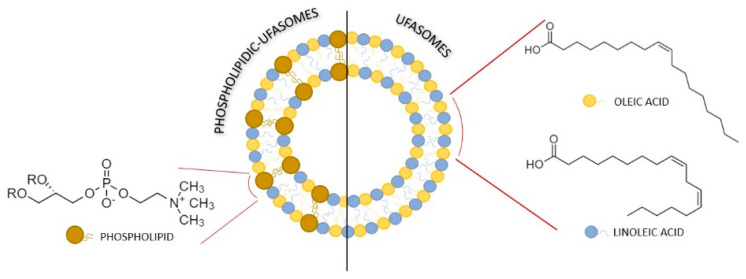
Structural model of a self-assembled ufasomes made of phospholipids and/or unsaturated fatty acids.

Figure 3 describes the hypothetical arrangement of unsaturated fatty acids and phospholipid, forming ufasomes structure.

Some important chemical-physical parameters that must be assessed during the design of new carriers are mean size, polydispersity index and zeta-potential. These features broadly affect the pharmacokinetic properties of ufasomes and their biopharmaceutical properties of delivered active substance.

The formulations A and B were submitted to dynamic light scattering analysis and, as can be seen in Table 2, both formulations showed mean size less than 300 nm and a narrow size distribution as demonstrated by the low polydispersity indices. Formulation A and B presented strongly negative zeta potential values in according with Morigaki et al. [22], due to the presence of lipophilic surfactants with negative charge, as PL90G, and fatty acid. These zeta potential values should guarantee repulsion between carriers, reducing the probability of instability phenomena. 

**Figure 4:**
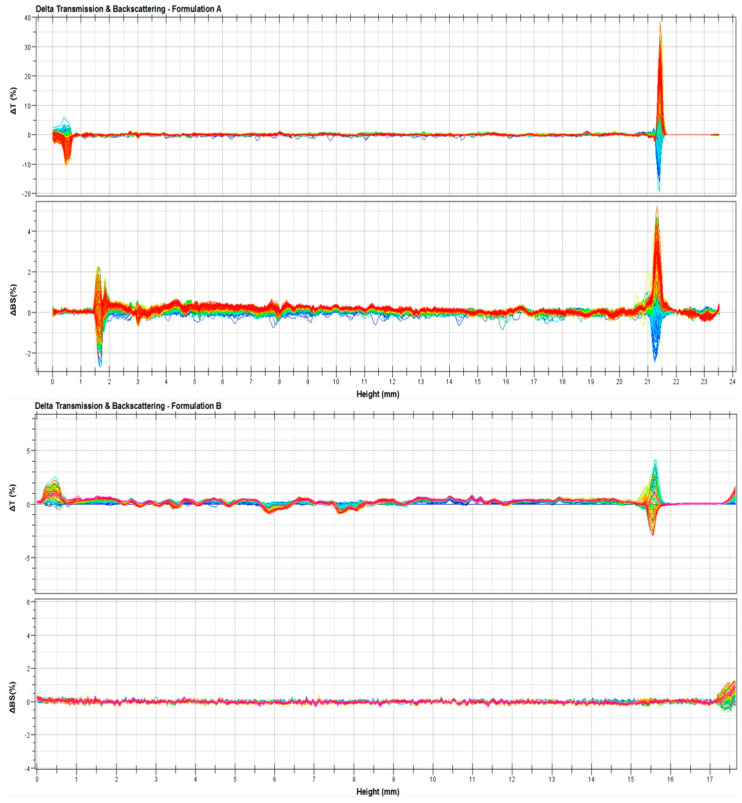
Delta transmission (ΔT) and delta back scattering (Δbs) profiles of (up) formulation A and (down) formulation B. Panels report representative experiments of three independent experiments. Data are reported as a function of sample height.

To confirm the hypothesized stability of the ufasomes, further studies were carried out by Turbiscan Lab^®^ Expert by measuring the backscattering and transmission profiles of systems as a function of time (0–1 h) and sample height (mm). Thank to this technique, any kind of possible destabilization phenomena was detected [41]. As shown in Figure 4, ∆t and ∆bs profiles remained in a narrow range during all the analysis time, and in particular the variation of profiles was within values of ±5%, thus indicating that no aggregation, creaming, flocculation or sedimentation phenomena occurred. In fact, Celia et al. [41] affirmed that only a variation of backscattering profiles greater than 10% is indicative for the presence of segregation phenomena. The variation in ∆t and ∆bs profiles that occur below 2 mm and over 8 mm did not depend on destabilization of ufasomes, but it was due to air inclusion in the bottom and/or the top of Turbiscan Lab^®^ vials [30]. These results confirmed the stability of the systems previously provided on the basis of zeta-potential values.

### 3.2. In Vitro Cytotoxic Evaluation

**Figure 5:**
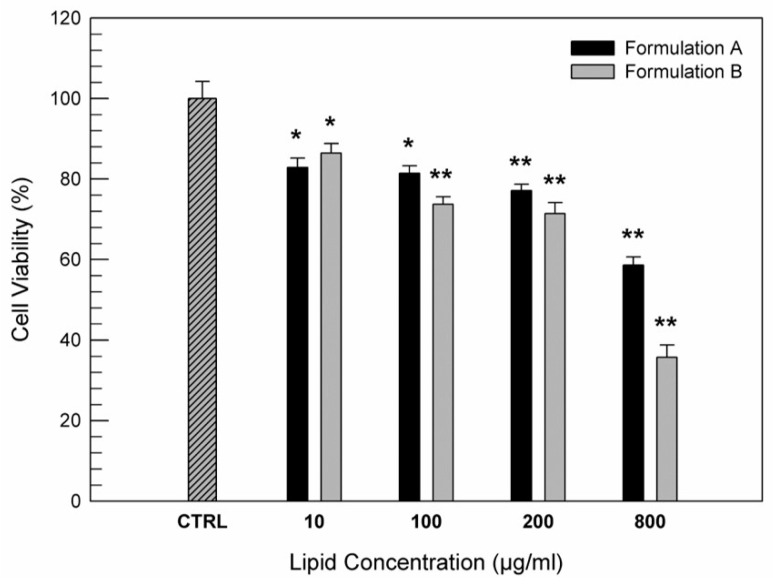
In vitro cytotoxic effects of Formulation A and Formulation B on CaCo-2 cells as a function of lipid concentration (µg/mL). The data are expressed as a percentage of cell viability after 24 h of treatments and they are the average of three different experiments ± standard deviation. * *p* < 0.05 and ** *p* < 0.001 versus control (untreated cells).

The biocompatibility of the structural natural components of ufasomes suggest that they can be administered into the human body [42,43]. In particular in this study, we proposed these systems as delivery systems for nutraceuticals, capable of treating any inflammatory and oxidative pathologies of the intestinal mucosa. For this purpose, CaCo-2 human colorectal adenocarcinoma cells were used as cellular model of intestinal tissue for testing the safety of Formulation A and B. MTT assay was carried out to evaluate the cell viability after 24 h of treatment with increasing concentrations of formulations. Figure 5 shows an appropriate safety indicating a good biocompatibility of both formulations with Caco-2 cells up to lipid concentration of 200 µg/mL, in fact the cell viability was kept above 70% respect to untreated cells (used as control). On the contrary, at higher lipid concentrations, especially the formulation B made of only unsaturated fatty acid induced a marked reduction in cell viability. Probably this cytotoxic effect on adenocarcinoma cells was due to the presence of oleic acid that at high concentrations seems to be able to induce an anti-tumoral activity on colon cancer cells, as already demonstrated by some research groups. In particular, some scientists demonstrated the ability of oleic acid, contained in nutraceutics, to inhibit colon cancer cell growth [43], confirming also the hypothesis that oleic acid represents an important nutrient for full reconversion of cancer cells into healthy intestinal cells [44]. These in vitro results are very encouraging because of the modulate effects of oleic acid as a function of its concentration.

### 3.3. Ufasomes-Cell Interaction

**Figure 6:**
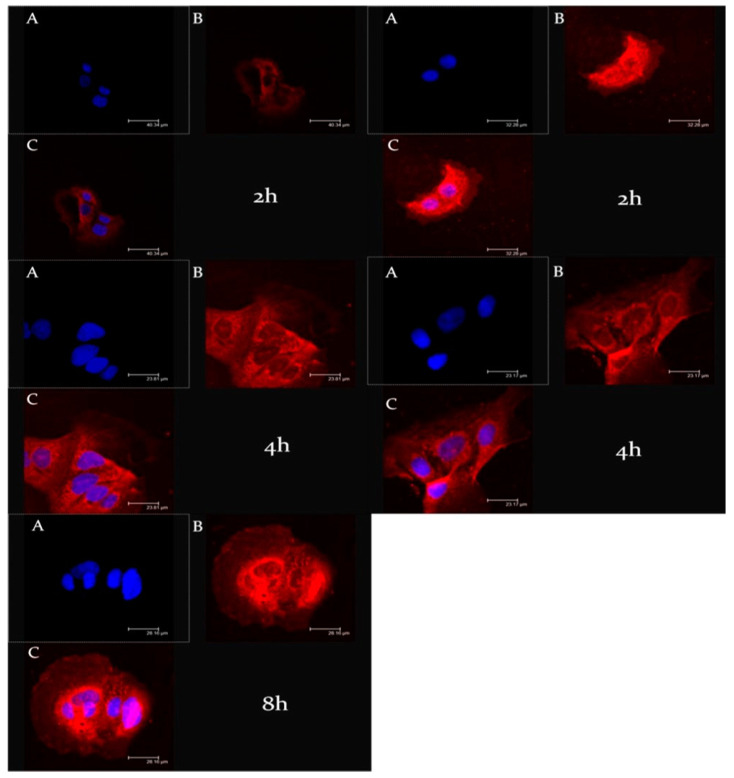
CLSM micrographs of CaCo-2 human colorectal adenocarcinoma cells treated with rhodaminated formulation (A) (left) and (B) (right) as a function of incubation time. (**A**): Hoechst filter; (**B**): TRITC filter; and (**C**): Overlay.

Another fundamental step during the design and the study of new nanosystems was the evaluation of their ability to interact with biological substrates. To evaluate the cell uptake of ufasomes, CaCo-2 cells were treated with rhodaminated formulations A and B (rhodamine-DMPE) for different incubation times (2 and 4 h); and as shown in Figure 6, confocal laser scanning micrographs confirmed the ability of ufasomes to interact with cells and this significant interaction could be correlated with a probable increased pharmacological activity of entrapped oleuropein. In particular, formulation A and B were internalized inside the cytosol already after 2 h of incubation; in fact, significant staining of the cytosol was evident for both formulations. The rapid and effective cellular internalization of ufasomes could be related with the permeation ability of unsaturated fatty acid that can increase the cell membrane fluidity [45].

Moreover, observing Figure 6, we can appreciate a greater intensity of red fluorescence in CaCo-2 incubated with formulation B, probably due to the exclusive presence of unsaturated fatty acid in its chemical composition that improved the ufasomes-cell interaction. 

**Figure 7:**
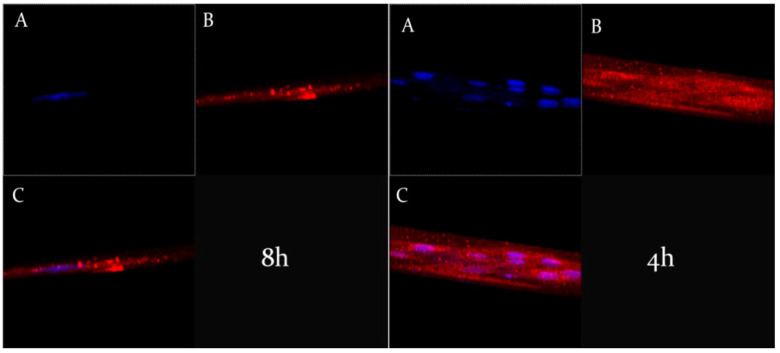
Z-stack analysis of CaCo-2 human colorectal adenocarcinoma cells treated with rhodaminated formulation (A) (left) and (B) (right), after 8 h and 4 h of incubation, respectively. (**A**): Hoechst filter; (**B**): TRITC filter; and (**C**): Overlay.

The Z-stack analysis, represented by Figure 7, confirmed the cytosolic localization of ufasomes and clearly demonstrated the integrity of nanocarriers structures inside the cells, due to a probable endocytosis-mediated processes [46]. 

### 3.4. Oleuropein-Loaded Ufasomes Preparation and Physico-Chemical Characterization

Starting from the promising results obtained in term of physico-chemical and technological-formulative characterization of formulation A and B, we decided to evaluate the ability of ufasomes to deliver the oleuropein, as natural compound characterized by antioxidant activity [9], and to improve its pharmacological activity on CaCo-2 human colorectal adenocarcinoma cells. 

For this purpose, the oleuropein-loaded formulation A and formulation B were prepared using two different active substance concentrations (10 µM and 100 µM) and were analyzed. The dynamic light scattering analysis of formulation A showed that increasing concentration of oleuropein induce a slight and progressive increase of mean size (Table 3), probably due to the insertion of the lipophilic portion of oleuropein between lipid molecules in the bilayer of ufasomes [18], that increase the distance between them. On the contrary, the presence of increased oleuropein concentration does not seem to have led to significant variation in the zeta potential values, and polydispersity index remained in a suitable range of values. These findings confirmed that the oleuropein did not alter the structure of formulation A. 

**Figure 8:**
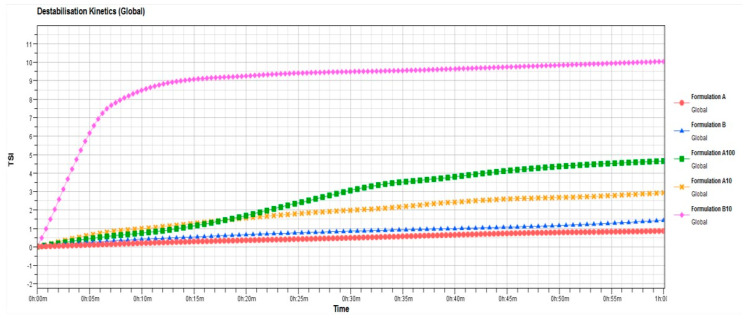
Turbiscan Stability Index (TSI) profiles of blank ufasomes and oleuropein-loaded ufasomes, obtained by Turbiscan Lab Expert^®^. The result was a representative of three independent experiments.

Contrary to formulation A, formulation B was shown to be affected by the presence of oleuropein already at the lowest tested concentration (10 µM). Probably, the composition of formulation B limited the effective encapsulation of lipophilic or amphiphilic active substances such as oleuropein. In our opinion, the mere presence of oleic acid and linoleic acid led to the formation of soft vesicles, which in the presence of high oleuropein concentrations are de-structured, leading to the formation of aggregates and the occurrence of instability phenomena as shown in the Figure 8. This marked difference in terms of stability and ability to contain the oleuropein between the two formulations was probably due to the presence of PL90G in the formulation A that elicits a stabilizing effect at the interface of ufasomes [47,48], enabling the nanocarrier to accept the active substance without undergoing changes in the bilayer structure.

In detail, Figure 6 confirms that formulation A well tolerates the presence of oleuropein at tested concentrations. In fact, stability kinetic profiles of formulation A10 and A100 fell within a narrow range of TSI (Turbiscan Stability Index); on the contrary, the profile of formulation B10 reached high TSI values, supporting the presence of instability phenomena.

Mean size, polydispersity index, zeta potential values and TSI profile of formulation B100 (100 µM of oleuropein) are not reported in Table 3 and in Figure 8 because the formulation underwent an evident sedimentation immediately after preparation. 

According to the data obtained through dynamic light scattering and Turbiscan Lab^®^ Expert analysis, the formulations A was chosen for oleuropein delivery and subsequent characterization studies of formulation A10 and A100.

### 3.5. Evaluation of Oleuropein Release Profile

Based on the previous studies, formulation A was chosen as the best ufasomes formulation for oleuropein delivery. The ability of formulation A10 and A100 to release the high encapsulated amount of oleuropein was investigated by using dynamic Franz cells and HPLC quantification. 

**Figure 9:**
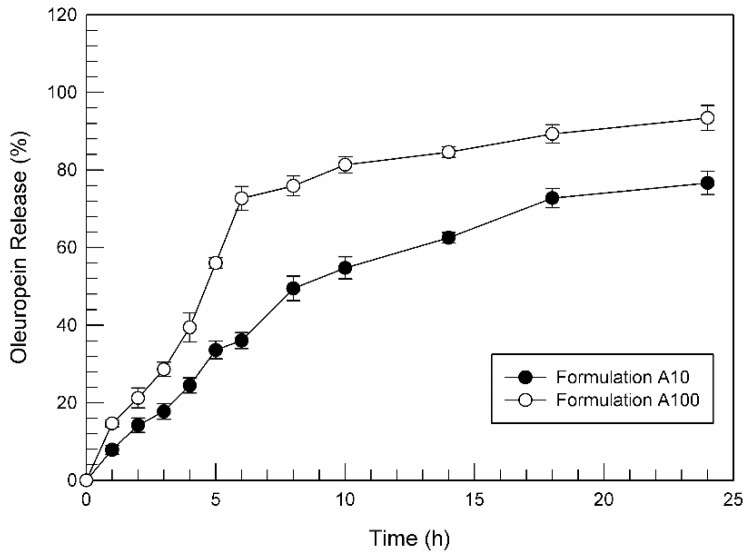
Release profile of oleuropein-loaded ufasomes. Values represent the mean of three different experiments ± standard deviation. If bars are not visible, they are within the symbols.

Figure 9 reports the results of performed in vitro release studies at body temperature (37 ± 0.1 °C) for formulation A10 and formulation A100. We can see that both formulations released more than 75% of entrapped oleuropein during 24 h. Moreover, a biphasic trend seems to characterize the release profile above all for the formulation containing oleuropein at 100 µM. That is, during the first 5 h of experiment, formulation A100 was able to release ~60% of entrapped active substance. An initial rapid oleuropein release from formulation can result in to very important and strategic since the ufasomes prepared in this study were shown to be able to internalize within the target cell in few hours, as shown by CLSM micrographs (Figure 4 and Figure 5). Observing the Figure 9, the release profiles of the two tested formulation seem to have a similar trend, even if formulation A10 kept lower values in terms of released oleuropein percentage, demonstrating the dependence of active substance release from entrapped concentration of oleuropein, as already demonstrated by other research groups for other nanosystems [20]. 

### 3.6. Antioxidant Activity Evaluation: MTT and LDH Assay

**Figure 10:**
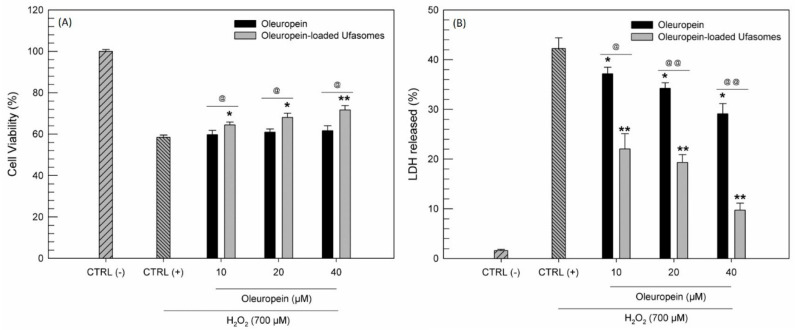
Antioxidant activity evaluation expressed as cell viability (**A**) and lactic hydrogenase (LDH) released (**B**). The tests were carried out on CaCo-2 treated with increased oleuropein concentrations, in free form and as active substance loaded ufasomes (Formulation A100), for 24 h and then with H_2_O_2_ (700 µM) for 1 h. The results are the average of three independent experiments ± standard deviation. * *p* < 0.05 and ** *p* < 0.001 versus H_2_O_2_; @ *p* < 0.05 and @@ *p* < 0.001 oleuropein-loaded ufasomes versus the same concentration of free oleuropein.

Our study was based on the idea of using a little-known type of nanosystems to improve the effectiveness of nutraceuticals such as oleuropein. The potentiality of oleuropein as natural active substance was amply studied and several research groups have demonstrated its ability to protect cells from oxidative stress [14,15]. For this reason, we decided to evaluate if the delivery of oleuropein by using ufasomes was useful to improve the antioxidant activity of free oleuropein. This evaluation was carried out by MTT test and LDH assay on CaCo-2 cell line. In detail, the cells were treated for 24 h with increased oleuropein concentrations in free form and as oleuropein-loaded ufasomes (Formulation A100), choosing the ufasomes composed by PL90G, oleic and linoleic acid, as best formulation. After this treatment, hydrogen peroxide was used to induce an oxidant stress on the cells. The results in terms of cell viability and LDH assay were reported in Figure 10. 

First of all, as already demonstrated by other research groups [49,50] we confirmed that CaCo-2 were very susceptible to the oxidizing action of H_2_O_2_, in fact when the cells were treated with H_2_O_2_ alone, a LDH release more than 40% and a reduction of cell viability equal to almost 40% were recorded. On the contrary, pre-treatment with oleuropein was shown to protect cells from the oxidizing hydrogen peroxide, with a significant reduction in the release of LDH. In detail, at the maximum used concentration of free oleuropein, a reduction of released LDH equal to ~15% (Figure 10B). These findings were consistent with the scientific work of Saija et al. [12], which have hypothesized that oleuropein could act as scavenger of chain-propagating lipid peroxyl radicals.

Probably thank to the capability of tested ufasomes to interact with CaCo-2 as already demonstrated by CLSM analysis, the nanosystems have shown to be able to improve the antioxidant activity of oleuropein. Observing Figure 10B, we can note that oleuropein-loaded ufasomes at maximum tested concentration are three times more effective in reducing LDH release than the free oleuropein. Moreover, the ability of ufasomes to improve the pharmacological activity of oleuropein was confirmed by the absence of intrinsic antioxidant activity of ufasomes, showed in Figure S2 (Supplementary Materials).

The LDH assay results are confirmed by the cell viability profile obtained by MTT assay. Moreover, in this case, the ufasomes formulation containing oleuropein provided the best results in term of oxidative protection of cells, compared to the free form of oleuropein (Figure 10A). 

## 4. Conclusions

The obtained data highlighted the potentiality of ufasomes to act as effective delivery system for an interesting nutraceutical compound, oleuropein. The physico-chemical and technological characterization of the two proposed formulation demonstrated that a certain lecithin portion, together with oleic and linoleic acid, increases the stability of the ufasomes structure; in fact, the formulation made only by unsaturated fatty acid resulted extremely sensitive to the inclusion of other molecules such as oleuropein. The in vitro studies demonstrated, in addition to the absence of toxicity of the formulations, the great ability of formulation containing PL90G to interact with cell membrane and to be internalized into cell model; this interaction allowed to obtain a more efficient protective action of entrapped oleuropein on cells when they are exposed to oxidative stress induced by H_2_O_2_, compared to free active substance. These findings showed that ufasomes are able to improve natural antioxidant activity of oleuropein on cell model. 

So, we can conclude that this is an example of nanotechnology that can help the application of natural compounds included in “super functional foods” and nutraceutics categories, by offering numerous advantages such as controlled release, increased shelf-life and improved cell interaction of entrapped active substance.

## Figures and Tables

**Table 1:** Lipid composition of different ufasomes formulations.

Formulation	Oleuropein (µM)	Lipid Composition (Molar Ratio)		
		Oleic Acid	Linoleic Acid	PL90G^®^
A	-	1	1	0.5
A10	10	1	1	0.5
A100	100	1	1	0.5
B	-	1	1	-
B10	10	1	1	-
B100	100	1	1	-

**Table 2:** Physicochemical and technological characterization of ufasomes.

Sample	Lipid Composition (Molar Ratio)			Mean Size (nm)	Polidispersity Index	Zeta Potential (mV)
	Oleic Acid	Linoleic Acid	PL90G			
A	1	1	0.5	185 ± 2	0.18 ± 0.01	−43 ± 1
B	1	1	-	280 ± 5	0.21 ± 0.02	−41 ± 1

**Table 3:** Physicochemical and technological characterization of oleuropein-loaded ufasomes.

Sample	Mean Size (nm)	Polidispersity Index	Zeta Potential (mV)	EE (%)
A	185 ± 2	0.18 ± 0.01	−43 ± 1	-
A10	184 ± 1	0.21 ± 0.01	−39 ± 2	85 ± 1
A100	199 ± 1	0.25 ± 0.03	−42 ± 1	89 ± 2
B	280 ± 5	0.21 ± 0.02	−41 ± 1	-
B10	350 ± 6	0.84 ± 0.06	−38 ± 5	45 ± 1
B100	-	-	-	-

## Data Availability

The data presented in this study are available on request from the corresponding author. The data are not publicly available due to privacy.

## Supplementary Materials

The following are available online at https://www.mdpi.com/2079-4991/11/1/105/s1, Figure S1: CLSM micrographs of CaCo-2 human colorectal adenocarcinoma cells Panel A: TRITC filter; panel B: Hoechst filter; panel C: overlay. Figure S2: Antioxidant activity evaluation expressed as cell viability (A) and LDH released (B). The tests were carried out on CaCo-2 cells treated with blank Formulation A, using the same concentrations necessary to deliver oleuropein at 10, 20 and 40 µM, for 24 h and then with H2O2 (700 µM) for 1 h. The results are the average of three independent experiments ± standard deviation.
