# Supplementary material for: Oleuropein-Laded Ufasomes Improve the Nutraceutical Efficacy
Source: Nanomaterials (Basel). 2021 Jan 4;11(1):105. doi: 10.3390/nano11010105 (PMC7824463; doi:10.3390/nano11010105)
Supplement: Supplementary file 1 [file nanomaterials-11-00105-s001.pdf]

nanomaterials

Article

Oleuropein-Laded Ufasomes Improve the Nutraceutical Efficacy

Maria Chiara Cristiano [1], Francesca Froiio [1], Antonia Mancuso [2], Donato Cosco [2], Luciana Dini [3], Luisa Di Marzio [4], Massimo Fresta [2] and Donatella Paolino [1,*]

1 Department of Experimental and Clinical Medicine, University "Magna Græcia" of Catanzaro, 88100 Catanzaro , Italy; mchiara.cristiano@unicz.it (M.C.C.); f.froiio@unicz.it (F.F.)
2 Department of Health Sciences, University of Catanzaro "Magna Graecia", 88100 Catanzaro, Italy; antonia.mancuso@unicz (A.M.); donatococosco@unicz.it (D.C.); fresta@unicz.it (M.F.)
3 Department of Biology and Biotechnology "Charles Darwin", Sapienza University of Rome, 00185 Rome, Italy; luciana.dini@uniroma1.it
4 Department of Pharmacy, University of Chieti-Pescara "G. d'Annunzio", 66100 Chieti, Italy; luisa.dimarzio@unich.it
* Correspondence: paolino@unicz.it; Tel.: +39-0961-369-4211

Abstract: Ufasomes are unsaturated fatty acid liposomes made up of oleic and linoleic acids, natural components required in various biological processes. This kind of nanocarrier is characterized by a simple and dynamic structure and is able to improve the bioavailability of unsaturated fatty acids. The aim of this investigation was to evaluate ufasomes as natural compound delivery systems to deliver oleuropein and improve its antioxidant activity. Oleuropein is a phenolic compound mainly present in olives and olive oil, with several biological properties, such as the antioxidant activity. However, to improve their biological activity, antioxidant compounds should be able to cross cell membranes and uniformly incorporate in cells. Because of the great similarity between their constituents and cell membranes, ufasomes could be advantageous carriers for oleuropein delivery. The physico-chemical characteristics of ufasomes were investigated. A regular shape was shown by transmission electron microscopy studies, while the mean sizes were dependent on the ufasomes composition. In vitro studies highlighted that empty ufasomes did not lead to cell mortality at the tested concentrations and a good carrier internalization in CaCo-2 cells, further studies in vitro studies demonstrated that oleuropein-loaded ufasomes were able to enhance the antioxidant activity of the free active substance making this carrier a suitable one for nutraceutical application.

Keywords: ufasomes; linoleic acid; oleic acid; oleuropein; antioxidant activity; unsaturated fatty acid liposomes

Citation: Cristiano, M.C.; Froiio, F.; Mancuso, A.; Cosco, D.; Dini, L.; Di Marzio, L.; Fresta, M.; Paolino, D. Oleuropein-Loaded Ufasomes Improve the Nutraceutical Efficacy. *Nanomaterials* **2021**, *11*, 105. https://doi.org/10.3390/nano11010105

Received: 30 November 2020
Accepted: 30 December 2020
Published: 4 January 2021

Publisher's Note: MDPI stays neutral with regard to jurisdictional claims in published maps and institutional affiliations.

Copyright: © 2020 by the authors. Submitted for possible open access publication under the terms and conditions of the Creative Commons Attribution (CC BY) license (http://creativecommons.org/licenses/by/4.0/).

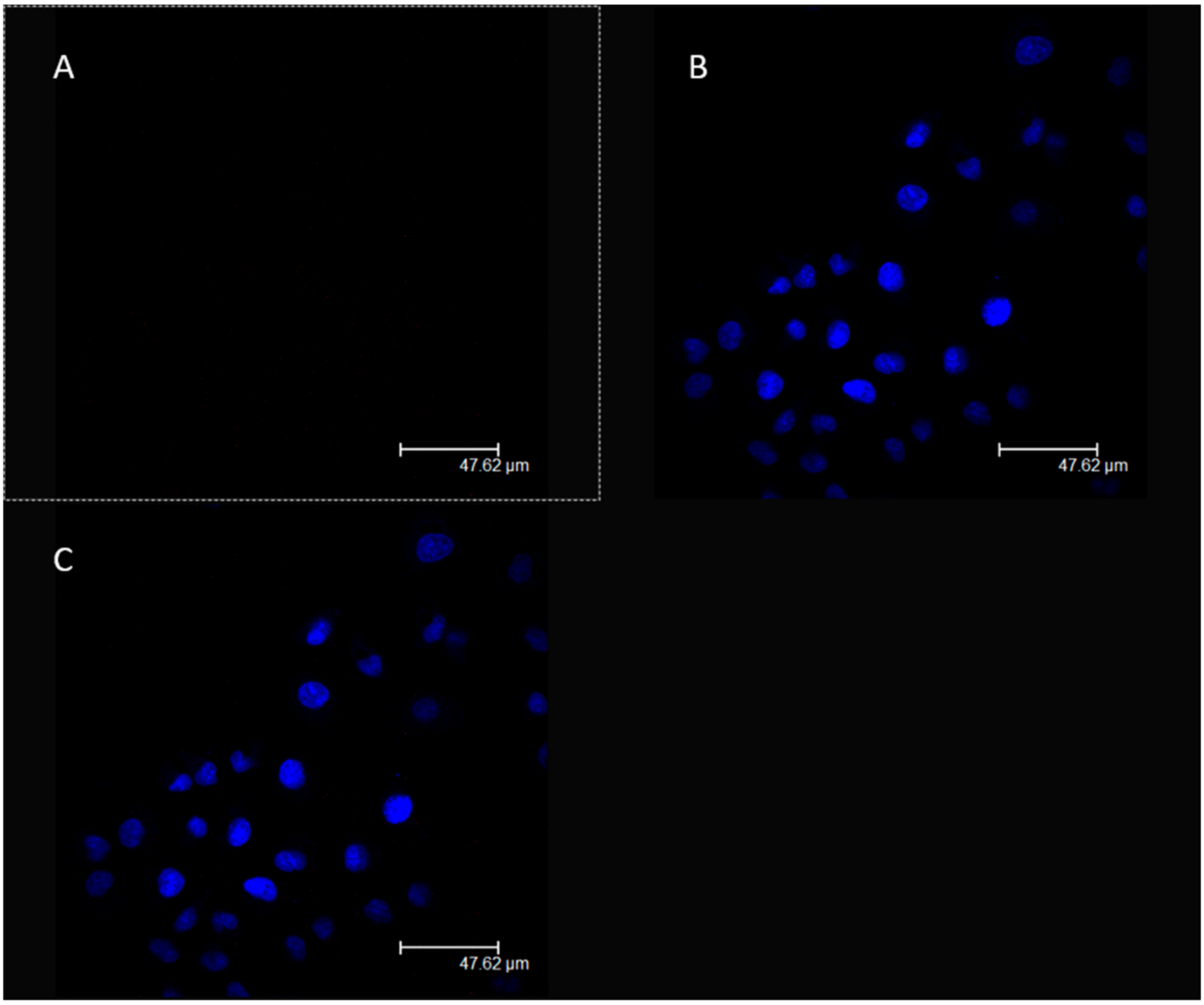

Figure S1. CLSM micrographs of CaCo-2 human colorectal adenocarcinoma cells. Panel **A**: TRITC filter; panel **B**: Hoechst filter; panel **C**: overlay. No autofluorescence phenomenon was observed.

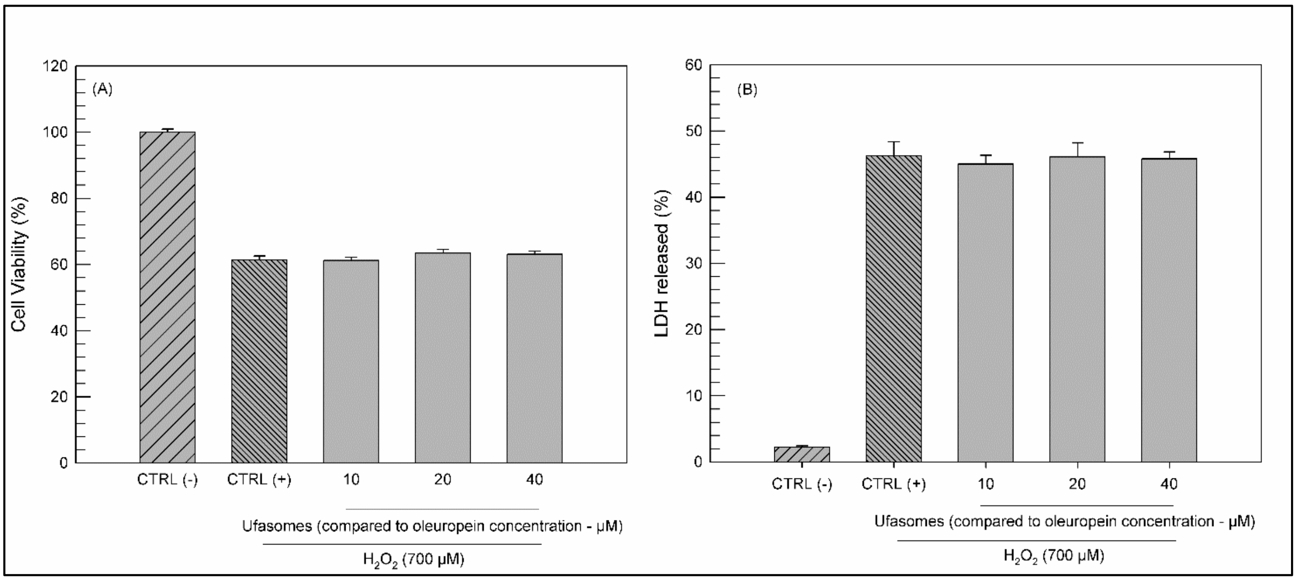

Figure S2. Antioxidant activity evaluation expressed as cell viability (**A**) and LDH released (**B**). The tests were carried out on CaCo-2 cells treated with empty Ufasomes (Formulation A), using the same concentrations necessary to deliver oleuropein at 10, 20 and 40 µM, for 24 h and then with H_2O_2 (700 µM) for 1 h. The results are the average of three independent experiments ± standard deviation. No significant antioxidant effect of blank formulation A was obtained. All results are not statistically significant compared to CTRL (+).
